# First days at sea: depicting migration patterns of juvenile seabirds in highly impacted seascapes

**DOI:** 10.7717/peerj.11054

**Published:** 2021-05-11

**Authors:** Maite Louzao, Karine Delord, David García, Isabel Afán, José Manuel Arcos, Henri Weimerskirch

**Affiliations:** 1 AZTI, Marine Research, Basque Research and Technology Alliance (BRTA), Pasaia, Spain; 2 Centro Oceanográfico de Xixón, Instituto Español de Oceanografía, Xixón, Spain; 3 Centre d’Etudes Biologiques de Chizé, UMR 7372 CNRS/ULR, Villiers-en-Bois, France; 4 Iniciativa de Recerca de Biodiversitat de les Illes (IRBI), Pina, Balearic Islands, Spain; 5 Estación Biológica de Doñana, Sevilla, Spain; 6 SEO/BirdLife, Barcelona, Spain

**Keywords:** Migratory behaviour, Juvenile movement ecology, Endangered species, Balearic shearwater, Fisheries, Cumulative human impacts

## Abstract

Increasing human activities have detrimental consequences on marine ecosystems and their impact can have cumulative effects. Within marine ecosystems, seabirds respond to ecosystem variability and face multiple human pressures, especially threatened species. In long-lived species, juveniles and immatures could represent up to 50% of the total population, but their migratory movements remain largely unknown. Here, we depict the migratory patterns of juvenile Balearic shearwaters *Puffinus mauretanicus*, the most threatened European seabird, using miniaturised satellite transmitters. At the end of the 2012 breeding season, five tagged juveniles left the breeding colonies of Eivissa Island (western Mediterranean) the first week of July. They moved westwards to reach the Atlantic Ocean between 3 and 13 days afterwards. Juveniles showed a two-phase migratory pattern: they first travelled slower close to the breeding colonies, and then moved towards their wintering areas in the Atlantic Ocean by rapid directional movements. Environmental cues (e.g.,marine productivity, water mass distribution, frontal systems) might have a prominent role in driving the migratory patterns of juvenile Balearic shearwaters, moving from warm and poor marine areas in the Mediterranean Sea to cooler and rich non-breeding grounds in the Atlantic Ocean. Based on observational findings, we observed certain spatial overlap of juvenile Balearic shearwaters with areas of high human impact, but the relationship between flying travel speed and both fishing effort and cumulative human impacts were not statistically significant. These results suggest that more research is needed to assess whether the movement patterns of migrating juveniles are affected by human activities. Therefore, understanding the at-sea spatial ecology of juveniles should be a priority for research and conservation due to the importance of this population component in long-lived species, as well as assessing their vulnerability to multiple anthropogenic pressures.

## Introduction

Human pressures are increasing globally in almost all types of ecosystems and national jurisdictions (Halpern et al., 2015). Human pressures have detrimental consequences on marine ecosystems and their impact can have cumulative effects  (Halpern et al., 2008; Halpern et al., 2015). Within marine ecosystem components, marine apex predators (e.g., seabirds) respond to ecosystem variability due to their position at the top of the food web (Heithaus et al., 2008). Seabirds are easy to monitor due to their land-based breeding. However, they face multiple human pressures on land including introduction of alien invasive species, hunting/trapping and disturbance (Dias et al., 2019). They also face threats at-sea including incidental bycatch in fisheries, overfishing and climate change (Dias et al., 2019). Most seabirds (∼70% of species, especially globally threatened) face multiple threats and pelagic seabirds represent the most threatened groups (Dias et al., 2019). Therefore, developing effective management and conservation measures for species requires the consideration of multiple threats through their lifetime (Carneiro et al., 2020).

Seabirds show fluctuating activity patterns during their annual cycle which are shaped by their breeding phenology and are linked to environmental cues and energetic requirements. For example, most seabirds have a restricted home range during breeding due to the constraints of their land-based reproduction, but they may switch to long-distance migration during the inter-breeding phase as they are released from these constraints. Initially many of the research and conservation initiatives on migration ecology focused on the dispersive movements of breeding individuals (i.e., Delord et al., 2013; Phillips et al., 2016). However, the movement ecology of juveniles and immatures remains largely unknown for many species (Péron & Grémillet, 2013; De Grissac et al., 2016; Corbeau et al., 2020), despite the fact that juveniles and immatures could represent up to 50% of the total population in long-lived species (Gaillard, Festa-Bianchet & Yoccoz, 1998). The first days at sea are critical for juveniles since they have to start exploring the marine environment for the first time to find food by themselves (Mougin et al., 2000), a period when their mortality can be high (Daunt et al., 2007; Afán et al., 2019).

The number of studies exploring how juveniles adjust to their new environment has increased with the advent of tracking systems (De Grissac et al., 2016). The use of satellite transmitters allows tracking of the first movements of juveniles without needing to recover them (Péron & Grémillet, 2013; Grémillet et al., 2015; De Grissac et al., 2016; Afán et al., 2019). These studies, still scarce, focused mainly on exploring differences between juveniles and adults in terms of flight and foraging capacities (De Grissac et al., 2017). Recent studies also enhance our understanding of juvenile decision-making processes of leaving their colonies and exploring their new foraging environments (Péron & Grémillet, 2013). This is especially important for initial phases such as post-fledging migration in order to study their ecology and manage their populations effectively (Hazen et al., 2012).

The Balearic shearwater *Puffinus mauretanicus* is the most threatened seabird in Europe and is listed as critically endangered by the International Union of the Conservation of Nature, with an annual population decline of approximately 14% and an estimated time to extinction of 61 years (Genovart et al., 2016). The global breeding population, concentrated in the Balearic Islands (western Mediterranean), is estimated to be approximately 3,200 pairs (Genovart et al., 2016), with a global population of approximately 25,000 individuals (Arcos et al., 2012; Arroyo et al., 2016). The common non-breeding range of the species extends in the NE Atlantic from the Atlantic Morocco to the English Channel (Arcos, 2011). Adult breeders are found in four main Atlantic foraging areas (the Bay of Biscay, the Western Iberian shelf, the Gulf of Cadiz and the West of Morocco), coinciding with the most productive oceanic areas between July and October (Guilford et al., 2012; Meier et al., 2017; Pérez-Roda et al., 2017).

Little information is available on the juvenile component of this species in terms of population size, foraging and movement ecology. It is believed that immature survival is low (0.434, 95% CI [0.351–0.520]) for a Procellariiforme within current environmental conditions and levels of human activities (Genovart et al., 2016). Population modelling has highlighted the relevance of anthropogenic impacts in the rapid population decline, notably bycatch in fisheries contributing approximately to half of the mortality (Genovart et al., 2016). Furthermore, one of the first threats that juveniles of pelagic seabirds face during the initial stage after fledging is light pollution. Balearic shearwaters, which are nocturnal at the breeding colony and therefore fledge at night, are especially at risk because artificial lights may attract juveniles during the fledging period, which may cause them to ground on land (Rodríguez et al., 2015).

Here, we depicted the first stage of the spatial migratory behaviour of juvenile Balearic shearwaters by analysing their first days at sea. Our results provide the first description of the post-fledging journey of the juveniles of this endangered species, from the breeding colonies in the western Mediterranean to their main wintering grounds in the Atlantic Ocean. We characterised movement parameters of tracked juveniles through their longitudinal migratory journey to address (1) whether juveniles were able to adjust their travel speeds to the different geographical phases of the migration based on their passage along distinct marine regions. We expected to find lower travel speeds in the Mediterranean Sea corresponding to a likely initial phase exploring their new marine environment, in contrast to both the Alboran Sea and Atlantic Ocean when they would probably acquire the necessary skills to continue their migratory journey. (2) Likewise, we explored oceanographic characteristics through proxies of marine productivity patterns, water masses distribution and frontal systems. We tested whether juveniles leave their natal marine grounds to find productive waters in their wintering areas. (3) Within this frame, we explored the levels of human activities such as fishing effort and an overall index of cumulative human pressures (considering multiple stressors such as pollution, fishing and climate impacts encompassing 17 anthropogenic pressures; Halpern et al., 2008) that juvenile Balearic shearwater might have faced. Did Balearic shearwaters respond to levels of human impact by changing their movement patterns? Depending on the level of human impact, juveniles could be attracted to human activities (e.g., fishing) providing food resources (e.g., discards, baits) and therefore the flying travel speed would decrease.

## Material & Methods

### Migratory tracking data

Experiments comply with the current laws of the country and were performed under the permission CEP 26/2012 of the *Direcció General de Biodiversitat* (*Conselleria de Medi Ambient i Mobilitat, Govern de les Illes Balears*). The study was carried out on *Illa de sa Conillera*, *Reserves Naturals des Vedrà, es Vedranell i els Illots de Ponent* (west of Eivissa Island, western Mediterranean) between the 21st and 24th of June 2012. We tagged 5 juveniles and we took morphological measurements (e.g., head plus bill length, minimum bill depth) to determine sex based on a species-specific discriminant function based on biometry measurements (Genovart, McMinn & Bowler, 2003). Three individuals were females, while two were undetermined. We deployed 5g Argos satellite transmitters PTT (Platform Terminal Transmitters) solar panel with a duty-cycle of 48-hour off, alternating with 10-hour on (Microwave Telemetry, Columbia). PTTs were attached on the back feathers using solely Tesa Tape^®^ (Guilford et al., 2008). This method allows a rapid deployment of the tag and avoids a medium to long term impact on the bird (Gillies et al., 2020). The use of the Tesa Tape resulted in a short tag deployment. We assumed that the transmission ended when the PTTs fell off. The total mass of the devices ranged between 0.72% and 1.06% of the total body mass. Body mass was determined using a 700g Pesola (±10g) balance.

**Figure 1:**
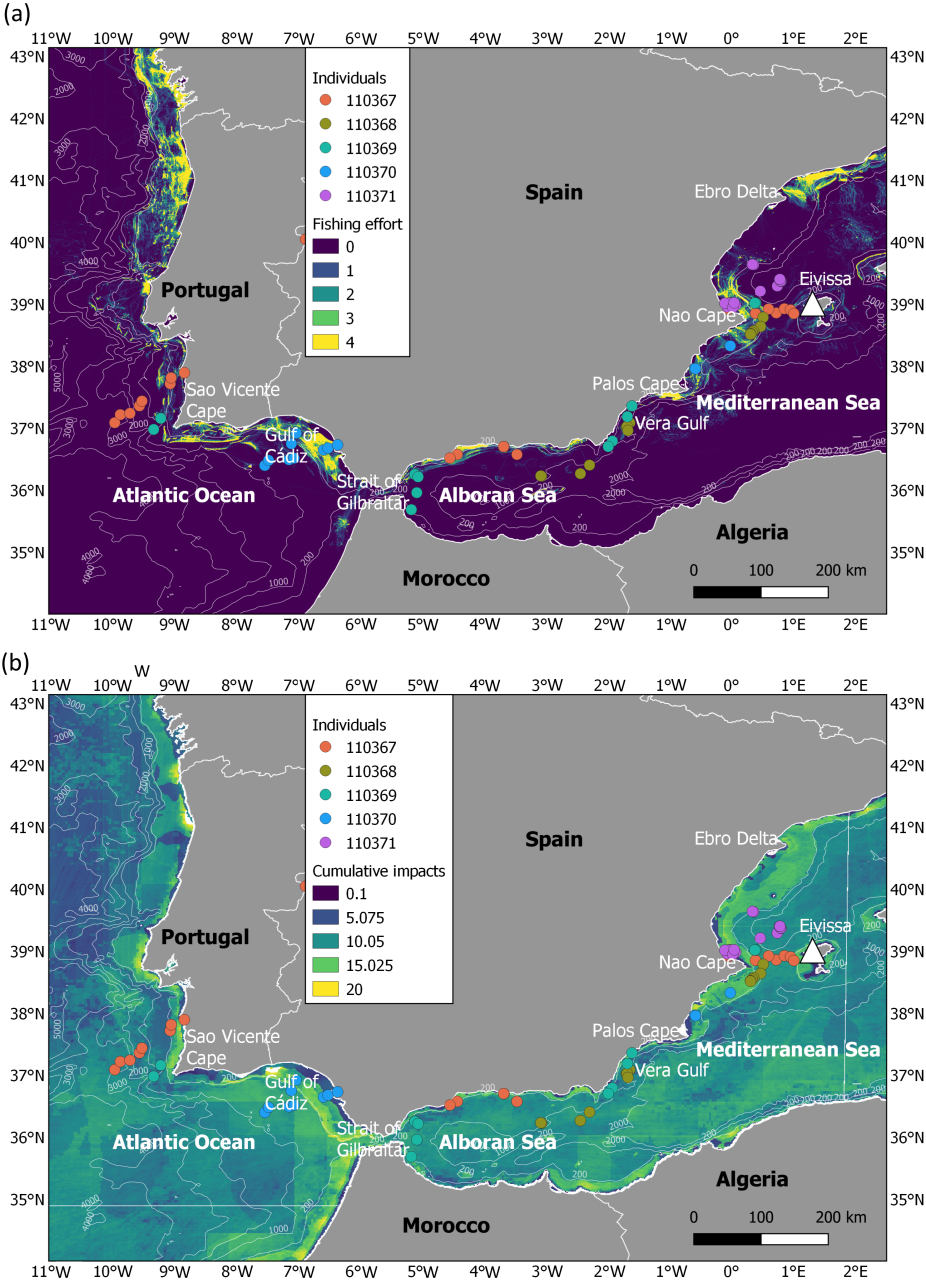
Migration of juveniles of Balearic shearwater in highly impacted seascapes. (A) Fishing effort (expressed as hours per square kilometre per month) and (B) cumulative human impacts (considering multiple stressors such as pollution, fishing and climate impacts encompassing 17 anthropogenic pressures). Fishing effort was extracted from the fishing density data developed within the EMODNET framework (https://www.emodnet-humanactivities.eu/documents/Vessel%20density%20maps_method_v1.5.pdf). Cumulative impacts from Halpern et al. (2008) were available at https://knb.ecoinformatics.org/view/doi:10.5063/F19C6VN5. The white triangle indicates the location of the breeding colony.

The Balearic shearwater performs a longitudinal migration from the breeding colonies in the Western Mediterranean, crossing the Alboran Sea, to their main wintering grounds in the Atlantic Ocean. The migratory journey is characterised by two oceanographic boundaries (the Vera Gulf and the Strait of Gibraltar), and therefore different parts of the journey take place under different oceanographic conditions. The western Mediterranean Sea and the Alboran Sea were divided by the Cape Palos (0.7°W), which is the northern limit of the Vera Gulf (Fig. 1). The Vera Gulf could be considered as an oceanographic boundary where waters of Atlantic origin meet Mediterranean surface waters forming important frontal structures such as the thermohaline Almeria-Oran front (Millot, 1999). Similarly, the Alboran Sea and the Atlantic Ocean were divided by the Strait of Gibraltar (longitudinal limit at 5.6°W) due to the distinct oceanographic conditions of both geographic domains, as they are considered isolated marine biogeographical regions (Borsa et al., 1997).

**Figure 2:**
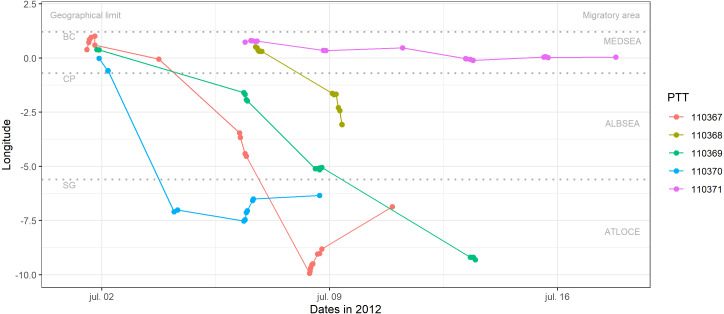
Longitudinal migration performed by juvenile Balearic shearwaters during their post-fledging dispersal. Each colour represents one individual (PTT number). Geographical limits are indicated as BC: breeding colony (Sa Conillera, SW Eivissa Island), CP: Cape Palos, SG: Strait of Gibraltar. Migratory areas are indicated as the Mediterranean Sea (MEDSEA), the Alboran Sea (ALBSEA) and the Atlantic Ocean (ATLOCE).

### Movement ecology

Before any analysis, we filtered the tracking data following Louzao et al. (2012). We first discarded positions over land and pooled all Argos locations (accuracy classes A, B, 0, 1 to 3). We then filtered positions above 70 km h^−1^ which is the maximum GPS-based speed of the closely related Manx shearwater *Puffinus puffinus* (Guilford et al., 2008), as done in previous studies (Louzao et al., 2012). This led to the removal of 10% of the positions. We estimated the travel speed between consecutive positions and a travel speed threshold of 10 km h^−1^ was used to distinguish flying from sitting behaviours. This speed threshold is based upon findings from previous studies on the ecology of shearwater species (e.g., Paiva et al., 2010). For each individual, we estimated: (i) the time between the date of deployment and the date of the first position at sea (PTTs could not transmit any position in the burrow) as an indicator of the time required to leave the colony; (ii) the date of passage through the Strait of Gibraltar calculated as the first position of PTTs when juveniles were located over the Atlantic Ocean; (iii) different movement patterns such as the mean (km h^−1^) and maximum travel speeds (km h^−1^) during the migration track, as well as the total distance covered (km) and the total duration of the transmission (days) as the time elapsed between the first and the last location recorded. The percentage of positions flying was estimated by diving the total number of flying positions with respect to the total positions of each individual over the migration track.

We explored changes in the movement parameters such as the travel speed and the percentage of flying by migratory area (from east to west): the western Mediterranean Sea (MEDSEA), the Alboran Sea (ALBSEA) and the Atlantic Ocean (ATLOCE). We applied Kruskal-Wallis tests to assess whether juveniles were able to adjust their travel speed and percentage of flying to the marine area crossed during the migratory journey. Significance level was set at 0.05.

### Oceanographic habitats through the migratory journey

We characterised the oceanographic habitats of each marine region visited by juvenile Balearic shearwaters during their first migratory journey. We explored their oceanographic habitats based on the most biologically relevant environmental variables from the known habitat selection of the species (Louzao et al., 2006; Louzao et al., 2011; Louzao et al., 2012). Environmental variables were obtained from the Environmental Research Division, Southwest Fisheries Science Center and US National Marine Fisheries Service (https://coastwatch.pfeg.noaa.gov/coastwatch/CWBrowserWW180.jsp?).

Amongst dynamic variables, sea surface temperature (SST; ^∘^C) and chlorophyll *a* concentration (CHL; mg m^−3^) are useful proxies of water mass distribution (prey types are reported to be associated with particular water masses; Hyrenbach & Veit, 2003) and ocean productivity (e.g., Le Fèvre, 1987; Longhurst, 1998), respectively. Spatial gradients of SST and CHL represent oceanographic fronts (i.e., marine areas where adjacent water masses of different properties come together) and are often characterised by convergence zones where marine organisms aggregate, including seabirds and their zooplankton and fish prey (Le Fèvre, 1987; Hunt et al., 1996). Previous studies in the western Mediterranean have shown an influenced of the spatial gradients of SST and CHL on the presence probability of Balearic shearwaters related to frontal systems over productive shelf waters and river plumes (Louzao et al., 2006; Louzao et al., 2012). Dynamic covariates were obtained for July 2012 from MODIS, as the monthly temporal resolution have provided reliable results for the study species and biogeographical area considered (Louzao et al., 2012). Spatial gradients were computed based on the spatial differences of the covariate of interest over the entire study area. Specifically, we estimated the Proportional Change (PC) within a surrounding cell grid within a 3 × 3 cell area using a moving window as follows: PC = [(maximum value − minimum value) *100/maximum value] (Louzao et al., 2012). Thus, a spatial gradient expresses the magnitude of change of a covariate and has no units. Coastal vs. pelagic domains were characterised by the bathymetry obtained from ETOPO1 and the spatial gradient of the bathymetry (BATG) was used as a proxy of the presence of topographic features (e.g., shelf break or seamount), estimated as explained previously (see above).

To depict the oceanographic habitats through their migratory journey, we extracted for each location and individual the values of monthly dynamic environmental variables (sea surface temperature, chlorophyll *a* and spatial gradients), as well as the bathymetry and the corresponding spatial gradient. We tested differences for the six environmental covariates by migratory area (MEDSEA, ALBSEA and ATLOCE). Kruskal-Wallis tests were applied to assess regional differences between the covariates. Significance level was set at 0.05. Finally, we tested whether the environmental values selected by juveniles differed from the available values within that geographical area (e.g., did juveniles use deeper waters in the Atlantic Ocean because of a choice, or simply because the Atlantic waters are deeper?). To do that, we compared the observed distribution of oceanographic variables selected by juveniles to the distribution of available oceanographic conditions. The available oceanographic conditions were extracted within a buffer of 250 km around the Iberian Peninsula, since this distance has been previously used as a search radius to identify potential foraging grounds of Balearic shearwaters (Louzao et al., 2011). In each migratory area, we randomly selected the same number of environmental covariate values available as juvenile observations, repeating the bootstrapping procedure 1000 times. For each iteration, a Kolmogorov–Smirnov test between the two distributions (oceanographic conditions selected by juveniles and available in each migratory area) was applied obtaining a distribution of statistics and *P-value*. After the bootstrapping procedure, the upper and lower 95% CI of the *P-value* distribution was obtained. If this distribution included the *P-value* of 0.05 (i.e., the 95% CI was not lower than 0.05), then there was no evidence that the environmental values selected by juveniles differed from the available values within that particular migratory area.

### Human pressures through the migratory journey

We characterised the level of human impacts in the migratory areas using two different sources of spatial information, 1) fishing effort and 2) cumulative human impacts (Fig. 1). While fishing effort (expressed as hours per square kilometre per month) was investigated as an indicator of the main threat for the Balearic shearwater at-sea (i.e., fishing bycatch) (Arcos, 2011), cumulative human impacts (considering multiple stressors such as pollution, fishing and climate impacts encompassing 17 anthropogenic pressures) were applied as a proxy of overall ecosystem-level pressure. We used the vessel density data for fishing developed within the EMODNET framework (https://www.emodnet-humanactivities.eu/documents/Vessel%20density%20maps_method_v1.5.pdf) (Fig. 1A). Since the oldest available data corresponded to 2017, we took the information of July 2017 as a relative proxy of fishing effort during our study period. We also extracted the cumulative human impacts estimated by Halpern et al. (2008) from https://knb.ecoinformatics.org/view/doi:10.5063/F19C6VN5 (Fig. 1B). Cumulative human impact information considers multiple stressors at a 1 km spatial resolution, with no units (more details in Halpern et al., 2008). The range of years considered to create the layer of each stressor can be checked in the Table S1 of Halpern et al. (2008); available at https://science.sciencemag.org/content/sci/suppl/2008/02/12/319.5865.948.DC1/Halpern_SOM.pdf. For each juvenile tracking position, we extracted the values of fishing effort and cumulative human impacts.

We tested differences on the levels of human impacts across the migratory journey by testing the spatial effect on the three distinct geographic domains (MEDSEA, ALBSEA and ATLOCE). Kruskal-Wallis tests were applied to assess regional differences between the the variables representing human impacts. Significance level was set at 0.05. Finally, we explored the response of juveniles to different levels of human impacts by means of individual activity. Specifically, we focused on the travel speed during flying to assess the response of juveniles to increasing levels of human activity. We developed Linear Mixed Models to test whether the travel speed during flying was influenced by levels of fishing effort or cumulative human impacts. For this purpose, the *lmer* function from the *lme4* package (Bates et al., 2015) was used. We adjusted a gaussian distribution to the travel speed with an identity link function. Fishing effort and cumulative human impacts were included as a continuous explanatory variable and ’Individual” was included as a random variable. The model formulation was: Travel speed = (1|Individual) + Fishing effort or Cumulative human impacts. The model with the fishing effort or cumulative human impact effects and the null model were compared by a likelihood ratio test using the *anova* function. The residual plots of the best models were investigated and no deviation from a linear form was detected.

## Results

### Movement patterns of migratory juveniles

Juveniles weighed on average 616.2 g (range: 470.0–695.0 g) during the fledgling period of 2012 (Table 1). One individual was equipped on the 21st of June 2012 and 4 birds on the 24th of June 2012. After deployment, 3 fledglings left the colony on the 1st of July 2012, whereas the two others left the colony on the 6th of July 2012, staying on average 10.46 days in the burrow (range: 7.76–12.63 days) (Table 2) before fledging. The number of locations obtained for juveniles ranged between 12 and 21 (Table 1). The total duration of the transmission (the time elapsed between the first and the last location recorded after PTTs fell off) was on average 8.36 days (range: 2.66–11.63 days) (Table 2).

**Table 1:** Summary of deployments of PTT devices on juvenile Balearic shearwaters and transmission characteristics. LOC number: number of locations.

PTT number	Weight (g)	Date of equipment	Date of first position	Date of last position	LOC number	Discarded LOC (%)
110367	656	21/06/2012	01/07/2012 12:59	10/07/2012 22:10	21	23.8%
110368	650	24/06/2012	06/07/2012 17:20	09/07/2012 9:13	14	0%
110369	695	24/06/2012	01/07/2012 20:18	13/07/2012 11:29	16	12.5%
119370	470	24/06/2012	01/07/2012 22:02	08/07/2012 16:35	12	8.3%
110371	610	24/06/2012	06/07/2012 9:36	17/07/2012 19:06	16	0%

**Table 2:** Summary of movement parameters of tracked juveniles of Balearic shearwater. Time to first location: time elapsed between the date of equipment and the date of first position at sea (= time required to leave the colony). Date of passage: the date of first Atlantic position after crossing the Strait of Gibraltar. The total duration of the transmission was the time elapsed between the first and the last location recorded.

PTT number	Time to first location (days)	Date of passage	Mean speed (km h^−1^)	Maximum speed (km h^−1^)	Total distance (km)	Total duration (days)
110367	10.45	08/07/2012	18.54	48.89	1570.93	9.38
110368	12.63	–	14.89	49.93	447.64	2.66
110369	7.76	13/07/2012	12.63	47.94	1148.49	11.63
110370	7.83	04/07/2012	10.96	28.74	864.30	6.77
110371	12.31	–	1.39	3.33	201.57	11.39

Three out of the five fledglings left the breeding colony the 1st of July 2012 and performed a westward migratory journey crossing the Strait of Gibraltar between 3 and 13 days (the 4th and 13th of of July 2012, Table 2) afterwards (Fig. 2). After crossing the Strait of Gibraltar, they stopped their westerly migratory journey, as indicated by the relatively low longitudinal changes in Fig. 2 (individuals 110370 and 110367). The individual number 110367 travelled the longest distance (more than 1500 km) with a higher mean speed (18.54 km h^−1^) during 9 days (Table 2). One individual (110368) reached the eastern Alboran Sea only 2.6 days after leaving the breeding colony (Fig. 2). Finally, the individual 110371 stayed in the Gulf of Valencia (during 11.4 days of transmission, between the 6th and 17th of July 2012) with a mean speed of 1.39 km h^−1^ and travelling a total of 201.6 km (Fig. 2, Tables 1 and 2).

**Figure 3:**
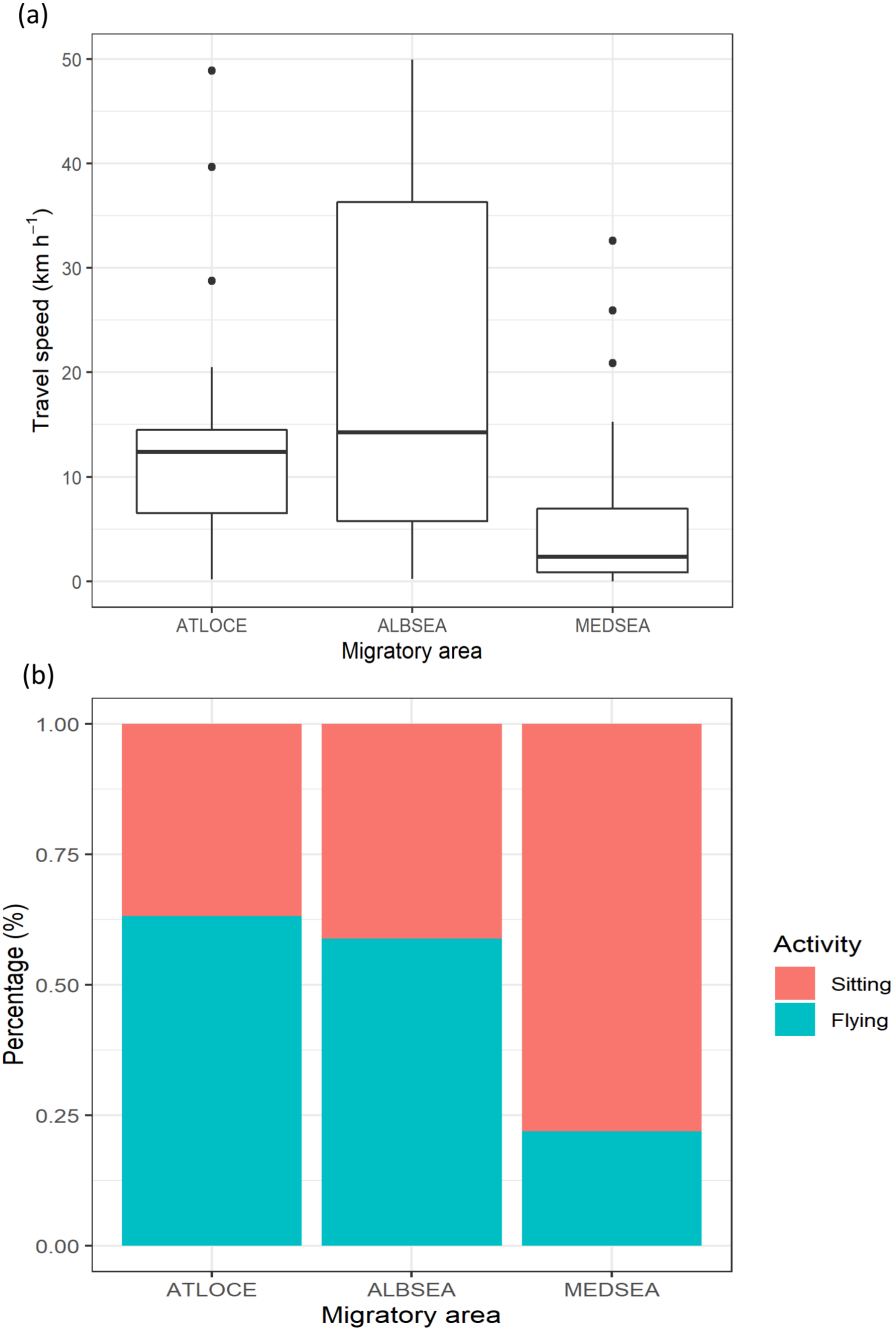
Movement patterns of juvenile Balearic shearwaters along their post-fledging migratory journey by means of (A) the travel speed (km h^−1^) and (B) the percentage of activity. The latter is the total number of flying or sitting positions with respect to the total positions in each migratory area. Mediterranean Sea: MEDSEA; Alboran Sea: ALBSEA; Atlantic Ocean: ATLOCE.

The travel speed of juveniles showed statistically significant differences between migratory areas (Kruskal-Wallis test: *H*_2,74_ = 14.21, *p-value* < 0.001). Travel speeds in the Mediterranean Sea (5.79 ±  7.95 km ^−1^) were lower than in both the Alboran Sea (19.85 ±  12.94 km ^−1^) and the Atlantic Ocean (14.02 ±  17.53 km ^−1^) (Fig. 3A). Similarly, the percentage of flying was lower in the Mediterranean Sea (21.87%) compared to the Alboran Sea (58.82%) and the Atlantic Ocean (63.15%, Fig. 3B).

### Oceanographic habitats used by migrating juveniles

**Figure 4:**
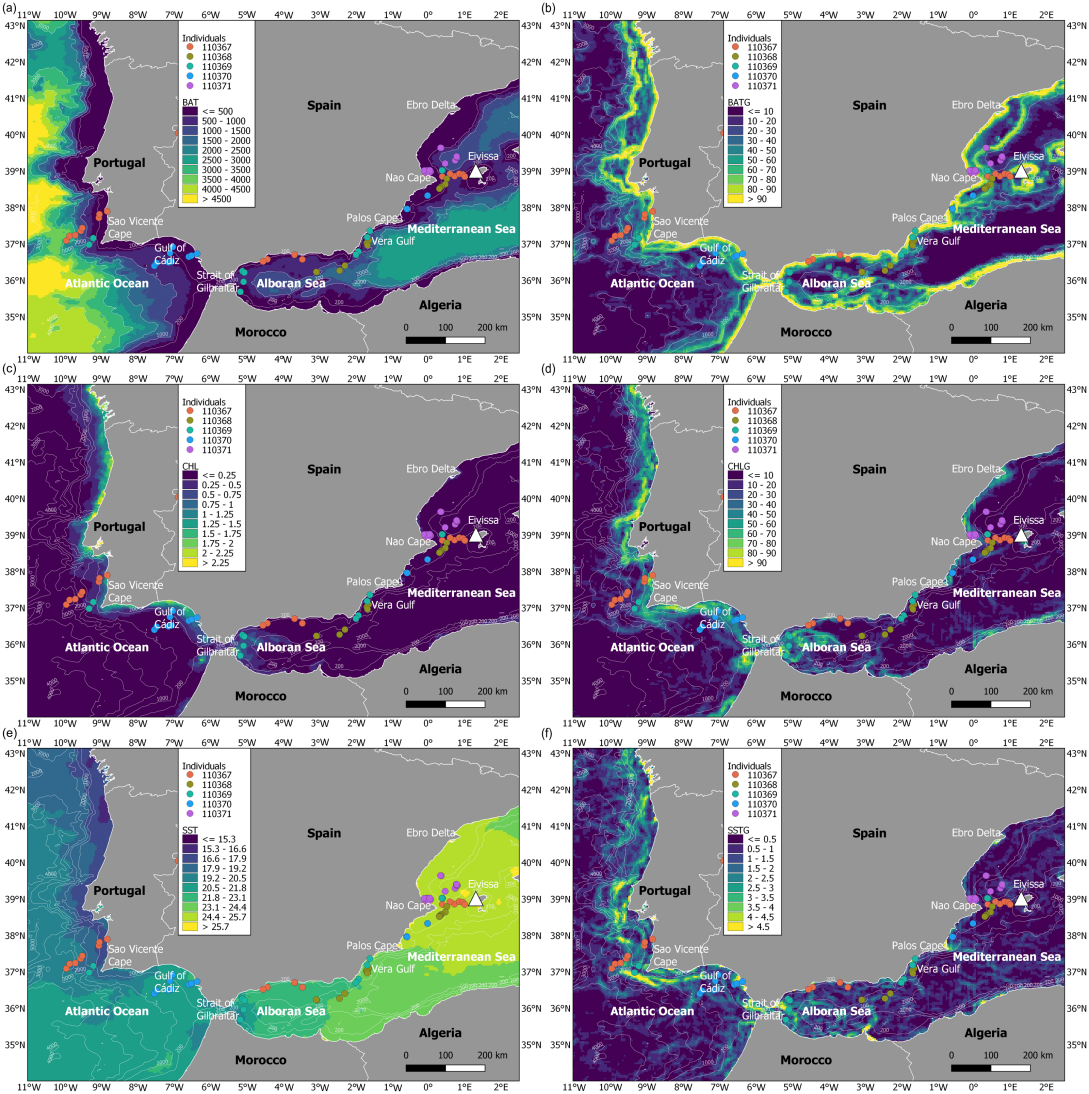
Maps of environmental variables through the migratory journey of juvenile Balearic shearwaters. (A) Bathymetry (m, BAT), (B) spatial gradient of bathymetry (no units, BATG), (C) sea surface chlorophyll *a* (mg m^−3^, CHL), (D) spatial gradient of chlorophyll *a* (no units, CHLG), (E) sea surface temperature (°C, SST) and (F) spatial gradient of sea surface temperature (no units, SSTG).

During the migratory journey, the oceanographic conditions were characterised by contrasting frontal systems in July (Fig. 4). The Atlantic Ocean presented stronger frontal systems (based on spatial gradients of both chlorophyll *a* and sea surface temperature) along the continental shelf, from the Strait of Gibraltar to the north of Spain, as marine productivity patterns were higher in this migratory area and sea surface temperature was lower and more heterogeneous. At an intermediate level, the Alboran Sea showed frontal systems of lower intensity, in contrast to the almost non-existent frontal systems in the Mediterranean Sea due to the more homogeneous chlorophyll *a* and sea surface temperature patterns. The bathymetrical ranges differed throughout the migratory journey of juvenile Balearic shearwaters: while the Atlantic Ocean showed higher ranges and more heterogeneous spatial patterns, the Alboran Sea and the Mediterranean Sea displayed lower bathymetrical ranges. The spatial gradients of the bathymetry indicated the location of coastal and continental slope areas, as well as submarine canyons and seamounts.

**Figure 5:**
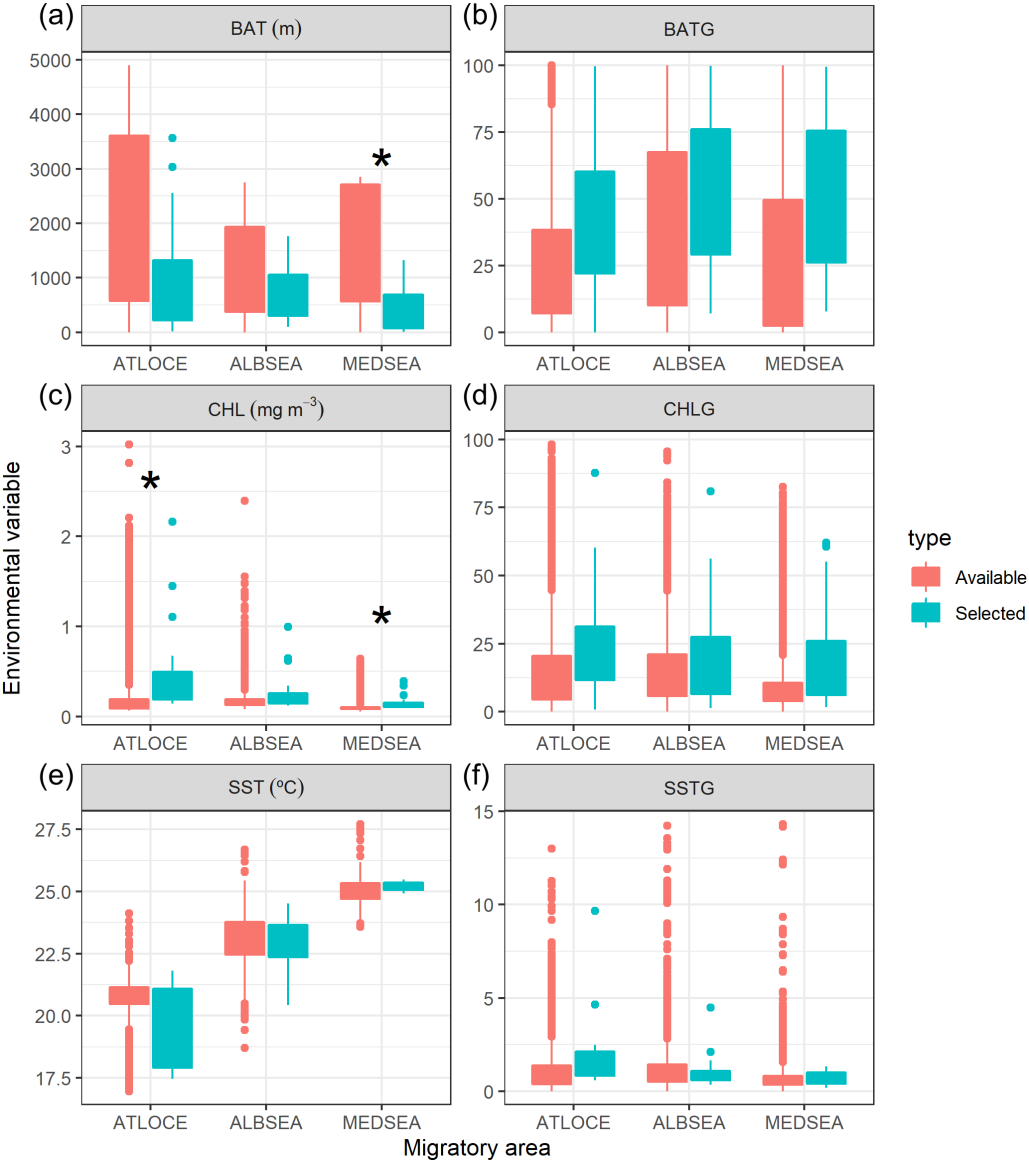
Boxplot of environmental variables selected by juvenile Balearic shearwaters crossing three different oceanographic areas during the migratory journey. From East to West: Mediterranean Sea (MEDSEA), the Alboran Sea (ALBSEA) and the Atlantic Ocean (ATLOCE). Statistically significant differences between selected and available environmental values are depicted by a black asterisk. (A) Bathymetry (m, BAT), (B) spatial gradient of bathymetry (no units, BATG), (C) sea surface chlorophyll *a* (mg m^−3^, CHL), (D) spatial gradient of chlorophyll *a* (no units, CHLG), (E) sea surface temperature (°C, SST) and (F) spatial gradient of sea surface temperature (no units, SSTG).

Oceanographic conditions selected by juvenile Balearic shearwaters were the following: the bathymetric values (Kruskal-Wallis test: *H*_2,74_ = 6.925, *p-value* = 0.031) increased from East to West (from the Mediterranean Sea, 439.32 ±  467.54 m, through the Alboran Sea, 731.67 ± 508.86 m, to the Atlantic Ocean, 984.53 ±  1038.78 m) (Fig. 5). The chlorophyll *a* also showed an increasing pattern from East to West: lower values and ranges in the Mediterranean Sea (0.14 ±  0.08 mg m^−3^), both intermediate values and ranges in the Alboran Sea (Sea 0.26 ±  0.22 mg m^−3^) and higher values and ranges in the Atlantic Ocean (0.51 ±  0.52 mg m^−3^) (Kruskal-Wallis test: H_2,74_ = 29.342, *p*-value < 0.001). The sea surface temperature decreased from East to West showing higher values within a narrower range in the Mediterranean Sea (25.20 ± 0.17 °C), compared to intermediate values and ranges in the Alboran Sea (22.83 ±  1.05 °C) and lower values and higher ranges in the Atlantic Ocean (19.63 ± 1.56 °C)(Kruskal-Wallis test: *H*_2,74_ = 61.712, *p-value* < 0.001) (Fig. 5). The spatial gradient of sea surface temperature showed an increasing pattern from East to West: lower values and ranges in the Mediterranean Sea (0.68  ±  0.36), both intermediate values and ranges in the Alboran Sea (1.04 ±  0.89) and higher values and ranges in the Atlantic Ocean (1.60 ±  0.95) (Kruskal-Wallis test: *H*_2,74_ = 18.076, *p-value* < 0.001) (Fig. 5). We did not find statistically significant differences between the spatial gradients of both bathymetry and chlorophyll *a* (Fig. 5).

We also tested whether the environmental values selected by juveniles differed from the available values within that migratory area (Fig. 5). The selection of environmental values by juveniles only differed statistically in the case of both the bathymetry and the chlorophyll *a* in the Mediterranean Sea and chlorophyll *a* in the Atlantic Ocean. No significant differences were found in the case of the spatial gradients of bathymetry, spatial gradients of chlorophyll *a*, sea surface temperature and spatial gradients of sea surface temperature selected by juveniles in relation to availability.

### Levels of human impact during migration

**Figure 6:**
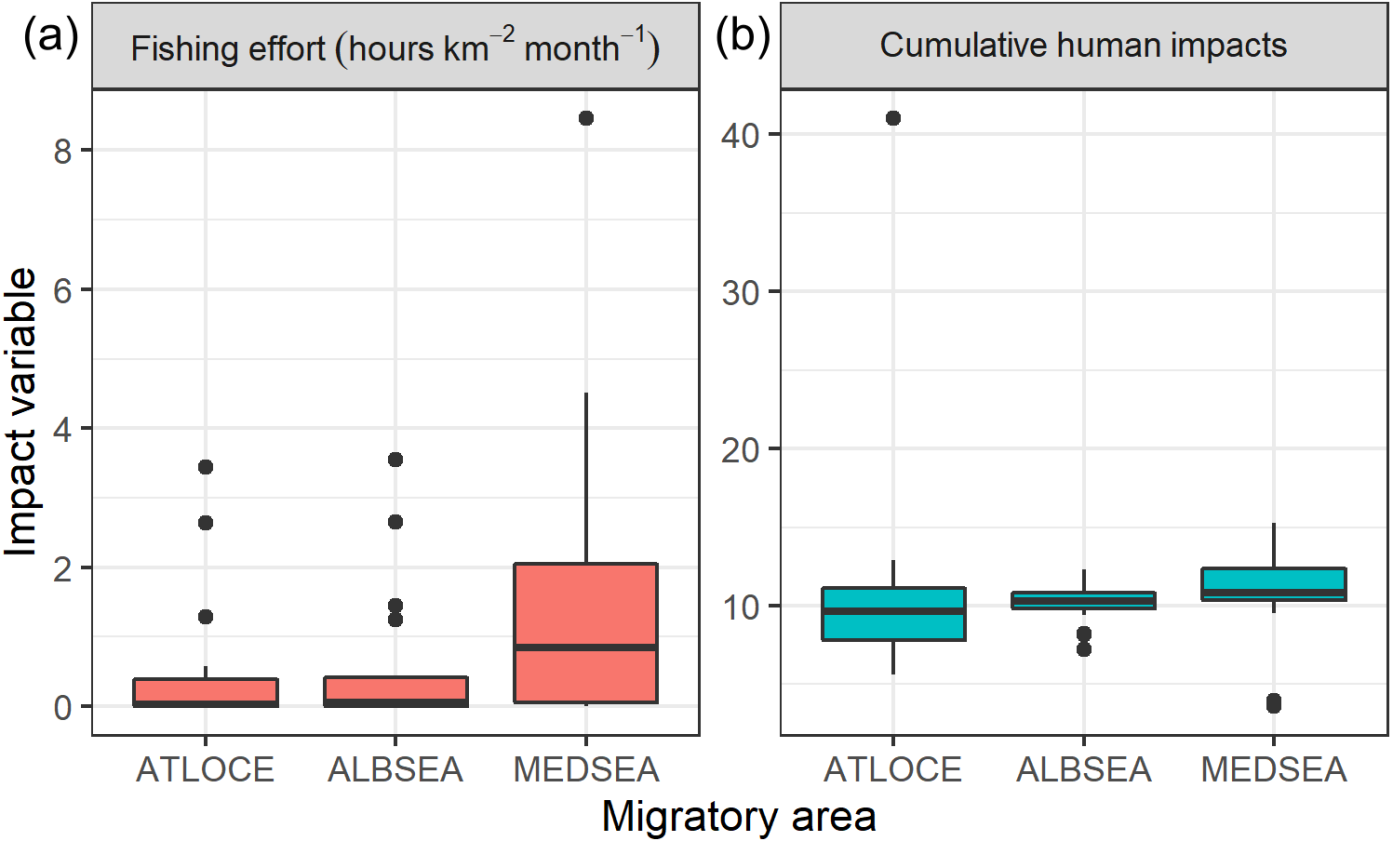
Boxplots of (A) fishing effort (hours km^−2^ month-1) and (B) cumulative human impacts (Halpern et al., 2008) through the migratory journey of juveniles Balearic shearwaters. From East to West: the Mediterranean Sea (MEDSEA), the Alboran Sea (ALBSEA) and the Atlantic Ocean (ATLOCE). From East to West: the Mediterranean Sea (MEDSEA), the Alboran Sea (ALBSEA) and the Atlantic Ocean (ATLOCE).

**Figure 7:**
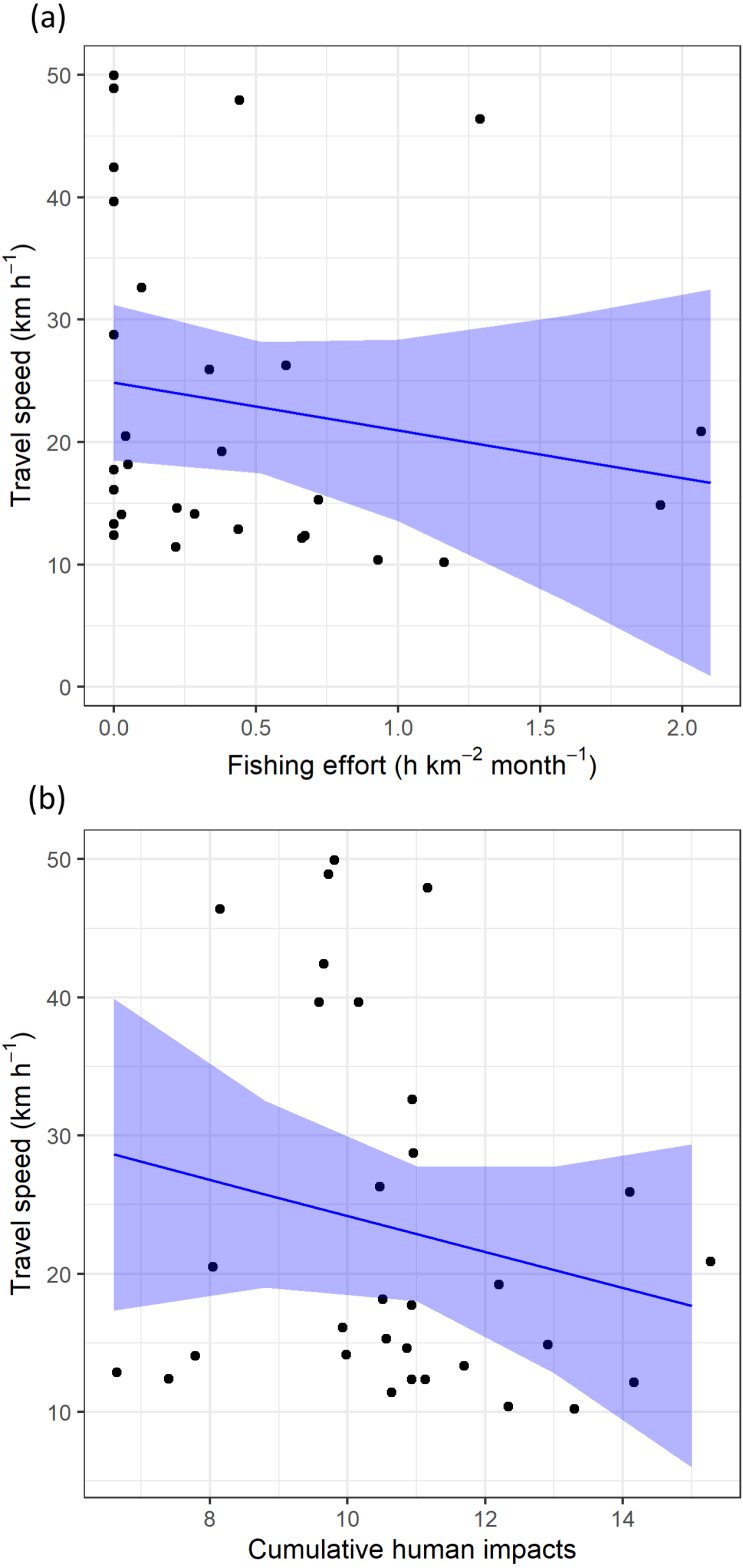
The travel speed of flying juvenile Balearic shearwaters according to (A) log-transformed fishing effort (expressed as hours per square kilometre per month) and (B) cumulative human impacts. The black dots represent the observed data while the blue lines represent the estimated values with Linear Mixed Models, while the shaded blue areas represent the interval confidence.

Regarding levels of fishing effort encountered by juveniles, fishing values were higher in the Mediterranean Sea (1.07 ± 1.20 h km^−2^ month^−1^) compared to values in the Alboran Sea (0.73 ±  1.20 h km^−2^ month^−1^) and the Atlantic Ocean (0.48 ±  0.96 h km^−2^ month^−1^) (Kruskal-Wallis test: *H*_2,74_ = 8.328, *p-value* = 0.015) (Fig. 6). On the other hand, values of cumulative human impacts did not differ between migratory areas (Kruskal-Wallis test: *H*_2,74_ = 5.154, *p-value* = 0.075), varying from 10.99 ±  7.55 in the Atlantic Ocean to 10.11 ±  1.27 in the Alboran Sea and 10.90 ±  2.89 in the Mediterranean Sea (Fig. 6). Fishing effort and cumulative human impacts were slightly correlated (*r*_*s*_ = 0.35, *p-value* = 0.019). When juveniles were actively flying, the travel speed decreased with increasing values of fishing effort even though this relationship was not statistically significant (${\chi }_{1}^{2}=0.942$, *P* = 0.331) (Fig. 7A). A similar pattern was found between decreasing values of flying travel speed and increasing levels of cumulative human impacts (${\chi }_{1}^{2}=1.208$, *P* = 0.271) (Fig. 7B).

## Discussion

In this work, we provide novel findings concerning the migratory ecology of the endangered Balearic shearwater, which takes place in one of the world’s most impacted seascapes: the Mediterranean Sea. We focused on a largely unknown population component depicting the movement ecology of juveniles during their first days at sea after fledging. These preliminary results are based on a small sample size and, therefore, conclusions should be considered as preliminary deserving further research. The juveniles, in an initial stage, left their natal colony and moved westward through the Mediterranean Sea and the Alboran Sea to reach the Atlantic Ocean. At the start, juveniles stayed in their burrow on average 10 days before leaving their breeding colonies (1st–6th of July 2012), coinciding with the known fledging period of the species during the first fortnight of July (Rodríguez et al., 2015). We captured the complete migratory journey (i.e., when birds travelled from the natal colony in the western Mediterranean to their main wintering grounds in the Atlantic Ocean) for only three of the juveniles who left earlier (1st of July 2012). Juveniles crossed the Strait of Gibraltar between 3 and 13 days after leaving the colony (the first fortnight of July). Since most adult breeders cross the Strait of Gibraltar at the end of June (ranging from late May to mid-July) (Guilford et al., 2012; Pérez-Roda et al., 2017), juveniles might follow similar migration patterns to that of adult breeders (Raine, Borg & Raine, 2011; Grémillet et al., 2015; De Grissac et al., 2017).

The travel speed and the percentage of flying of juvenile Balearic shearwaters changed during the different geographical phases of the migratory journey. They travelled slower close to the breeding colonies in the Mediterranean Sea possibly reflecting a mixture of increased exploratory and foraging behaviour, followed by an increase of the travel speed in both the Alboran Sea and the Atlantic Ocean. A similar two-phase pattern was identified in migrating juveniles, immatures and adults of Scopoli’s shearwaters *Calonectris diomedea*: a first phase in the Mediterranean Sea, followed by rapid directional movements towards wintering areas in the Atlantic Ocean (Péron & Grémillet, 2013). Juveniles of Scopoli’s shearwaters staged during a longer period in the western Mediterranean Sea in comparison to the other two age classes, probably to refill energy reserves due to low foraging abilities as has been described in raptor migration (Péron & Grémillet, 2013 and references therein).

It is widely accepted that seasonal changes in food resources and environmental conditions can drive animal migration (Dingle, 2014). Migratory movement due to seasonal variation in food resources is largely extended among pelagic seabirds (Shaffer et al., 2006). Therefore, environmental cues (e.g., marine productivity, water mass distribution, frontal systems) might also have a prominent role in driving the migratory patterns of juvenile Balearic shearwaters. When Mediterranean foraging grounds become warmer and less productive in early summer (Lazzari et al., 2012), juveniles (this study) and adults (Guilford et al., 2012; Pérez-Roda et al., 2017) may undertake migration to cooler and more productive Atlantic foraging areas. The common non-breeding range of the species extends in the NE Atlantic from the Atlantic Morocco to the English Channel (Arcos, 2011), coinciding with the most productive areas between July and October (Guilford et al., 2012; Meier et al., 2017; Pérez-Roda et al., 2017). Juveniles probably visit similar marine areas, as other Mediterranean pelagic seabirds breeding in the Mediterranean Sea and winter in the Atlantic Ocean (e.g., Péron & Grémillet, 2013), and presumably spend a remaining part of the year in the English Channel (Jones et al., 2014). Nevertheless, differences of habitat use could be expected between age classes with adults exploiting more restricted optimal environmental niches, as it is the case for the Scopoli’s shearwater (Péron & Grémillet, 2013). Our findings illustrate the initial phase of juvenile migration for the first time, but the limited tracking time of juveniles prevent us from depicting the whole migratory journey.

By using spatially explicit human activity information, we assessed the potential link between juvenile migration movement and human activities in one of the most impacted marine ecosystems in the world (Coll et al., 2010). Based on observational findings, we detected certain spatial overlap of juvenile Balearic shearwaters with areas of high human impact during their first migration (e.g., Nao Cape). The relationship between the travel speed of flying individuals and both fishing effort and cumulative human impacts were not statistically significant, and calls for further research to assess the likely attraction of naïve juvenile seabirds to human activities such as fishing (Afán et al., 2019). More detailed analyses of fishing activity discerning types of fishing gears could shed more light on whether the fishing gears exhibiting the highest levels of bycatch risk for the species (i.e., demersal longline and purse seine; Cortés, Arcos & González-Solís, 2017; Oliveira et al., 2015) are particularly affecting juveniles. This assessment would provide relevant information for specific conservation measures for the protection of the critically endangered Balearic shearwater.

Understanding the environmental adaptability and behavioural strategies to potential anthropogenic pressures can seriously impact the population dynamics of the endangered Balearic shearwater (e.g., Votier et al., 2008). Conservation efforts and management measures should be reflected at the spatial scale used by individuals, implying site-specific measures (Péron & Grémillet, 2013; Sherley et al., 2013). Spatially-explicit management measures such as Marine Protected Areas within the Natura 2000 network largely cover the main foraging grounds of adult breeders in Spain and Portugal (Pérez-Roda et al., 2017) and we could expect a similar coverage for juveniles. Nevertheless, alternative management efforts related to ecosystem-based fisheries management such as mitigation of fishery bycatch might be necessary to complement spatially-explicit measures (e.g., Sherley et al., 2013) within a transboundary context (Louzao et al., 2012). In a context of increasing multiple anthropogenic pressures, the analyses of cumulative human impacts can guide the development of required management strategies within an ecosystem-based framework (Halpern et al., 2015). At the European level, the Marine Strategy Framework Directive is a management tool that considers cumulative human impacts on marine ecosystems and additional legislation incorporates the Balearic shearwater as a priority species (e.g., Annex I of the Birds Directive, OSPAR Convention, Annex II of the Mediterranean SPA/BD Protocol) (Arcos, 2011). Consequently, depicting the year-round distribution of poorly documented population stages (juveniles and immatures) of endangered species should be prioritised to assess their vulnerability to multiple anthropogenic pressures (e.g., Clay et al., 2019).

##  Supplemental Information

10.7717/peerj.11054/supp-1 Supplemental Information 1 Raw positional data of juveniles of Balearic shearwaters

## Additional Information and Declarations

### Field Study Permissions

The following information was supplied relating to field study approvals (i.e., approving body and any reference numbers): Experiments comply with the current laws of the country, and were performed under the permission CEP 26/2012 of the Direcció General de Biodiversitat (Conselleria de Medi Ambient i Mobilitat, Govern de les Illes Balears).

### Data Availability

The following information was supplied regarding data availability: The data is available at BirdLife International: http://seabirdtracking.org/mapper/?dataset_id=1637.
